# Digesting the influence of diet and exercise on the microbiome

**DOI:** 10.1113/JP289733

**Published:** 2025-09-17

**Authors:** Simon Keely, Kerith Duncanson

**Affiliations:** ^1^ College of Health, Medicine and Wellbeing The University of Newcastle Callaghan New South Wales Australia; ^2^ National Health and Medical Research Council (NHMRC), Centre of Research Excellence in Transforming Gut Health The University of Newcastle Newcastle New South Wales Australia; ^3^ Immune Health Research Program, Hunter Medical Research Institute New Lambton Heights New South Wales Australia

**Keywords:** diet, exercise, microbiome, obesity

A host's diet and their microbiome are intrinsically linked, with the gut's microbial ecology developing in response to available nutrients sourced from food. Indeed, on a genetic and enzymatic level, the digestive capacity of the microbiome far exceeds that of a human and there is a growing view that traits of microbial digestion may contribute to the onset of some chronic diseases including obesity and metabolic syndrome. Obesity is a global health problem affecting over one‐third of adults worldwide. While genetic factors can play a role, the major driver of weight gain in overweight and obese individuals is excess energy intake through diet, relative to energy expenditure through physical activity. This imbalance drives weight gain at a metabolic level and sustained management of obesity is reliant on addressing this energy imbalance holistically (Taylor et al., 2019).

Given that the microbiome is largely responsible for the liberation of nutrients from our diet, there has been much research focus on the role of the microbiome in obesity and specifically whether some microbiomes may predispose individuals to weight gain. The majority of these studies have been associative, identifying changes at the phylum or family level and do not reflect the complexity of the gut microbial ecosystem (Duncanson et al., 2024). Further, while important, many clinical tools to evaluate diet are prone to inaccuracies (Taylor et al., 2019) and are not sufficiently detailed to provide information on micronutrient food composition to adequately predict the interaction of diet with the microbiome (Duncanson et al., 2024). In the case of obesity and weight loss, this is further complicated by the influence that exercise can have on the composition of the microbiome, both in terms of changes in metabolic physiology and through the gut‐brain axis (Clauss et al., 2021) and in many human studies, it is difficult to separate the influence of diet and exercise on the microbiome. In this sense the work by Davies et al. published in this issue of *The Journal of Physiology* offers interesting insights on the microbiome's role in weight loss mediated through energy restriction and exercise (Russell et al., 2025). Using a randomised controlled trial of intensive exercise coupled with restriction to dietary energy intake without changing nutrient proportions of the diet, they found that the microbial diversity remains stable despite improvements in body composition and metabolic health.

These data have some interesting implications for our current understanding of diet‐microbiome influence on obesity. For starters, they call into question the functional significance for the community profiles associated with overweight and obese individuals. While these profiles, typically with enriched Firmicutes and reduced Bacteroidetes, may develop with the dietary and metabolic changes that drive obesity (Magne et al., 2020), the data here suggest that these may be symptomatic rather than causal associations. Davies et al. performed energy restriction while maintaining the participants original diet, which would suggest that the microbiota's adaption to dietary composition isn't a significant driver of metabolic dysfunction. Because many studies on weight loss utilise changes in dietary composition (Duncanson et al., 2024; Taylor et al., 2019), it is interesting to speculate on how the change in diet, and the influence that change has on the microbiome as it adapts to the nutrient sources, confounds the results in this field.

There are several important caveats here, including that the Davies study does not provide information on the usual foods of participants and the extent to which individual dietary composition related to the magnitude of microbiome change. For example, if dietary fibre did not alter significantly, the expected change in microbiome composition change would be minimal. The intervention group were required to under‐consume their habitual diet by 5000 kcal/week, which would have still provided an average of 1500 calories/day. Without reporting of intervention period dietary intake relative to baseline, it is not possible to attribute causation to energy deficit alone. Accurate dietary assessment and analysis throughout interventions aimed at altering the gut microbiome are imperative to advancing understanding in this domain (Duncanson et al., 2024). Similarly, given the relatively short timing of the study, it remains to be seen whether the microbiomes were more resilient to change because they were still accessing their primary nutrient sources, and whether this just delayed more overt changes to microbial diversity.

The question remains of whether altered microbiomes are a cause or a consequence of obesity. Although diversity metrics here suggested stability in the microbiome across arms, a more in‐depth analysis of the functional metagenomic and transcriptomic pathways may indicate subtle but significant changes in the microbial ecology with increased metabolic health. Similarly, diet remains a confounder to studies of exercise and the microbiome (Clauss et al., 2021) and although the exercise benefits here are apparent, it would be reasonably expected that a nutritionally optimised diet and the presumed associative changes to the microbiome, would have increased the benefits to participants.

Major barriers to managing obesity include non‐compliance with and attrition from dietary and exercise regimens aimed at reducing weight. This is often driven by an aversion to ‘health food’ that is deemed important not only in managing calories, but also in restoring gut health. The data here, which indicated improvements in both body composition and metabolic indicators, suggest that lowering portions before wholesale compositional changes to diet, may be as effective and may be more palatable to the patients. Whether this approach could be maintained for longer durations and without the supervised support of a clinical study remains to be seen. Nevertheless, the tools to study diet‐microbiome interactions are still limited and fail to capture the complexity of the large ecosystem at play in the gut, but as our understanding increases and more datasets become available, more comprehensive strategies for managing metabolic health should emerge.

## Additional information

### Competing interests

No competing interests declared.

### Author contributions

S.K.: Conception or design of the work; Drafting the work or revising it critically for important intellectual content; Final approval of the version to be published; Agreement to be accountable for all aspects of the work K.D.: Conception or design of the work; Drafting the work or revising it critically for important intellectual content; Final approval of the version to be published; Agreement to be accountable for all aspects of the work.

### Funding

Federal Government | DHAC | National Health and Medical Research Council (NHMRC): Simon Keely, Kerith Duncanson, 2 035 319.

## Supporting information


Peer Review History
