# Supplementary material for: Digesting the influence of diet and exercise on the microbiome
Source: J Physiol. 2025 Sep 17;603(24):7765–6. doi: 10.1113/JP289733 (PMC12704029; doi:10.1113/JP289733)
Supplement: Supplementary file 1 — Peer Review History [file TJP-603-7765-s001.pdf]

The Journal of Physiology

Digesting the influence of diet and exercise on the microbiome

Simon Keely and Kerith Duncanson
DOI: 10.1113/JP289733

Corresponding author(s): Simon Keely (simon.keely@newcastle.edu.au)

The following individual(s) involved in review of this submission have agreed to reveal their identity: Dylan Thompson (Referee #1)

Review Timeline:

Submission Date:	11-Aug-2025
Editorial Decision:	20-Aug-2025
Revision Received:	27-Aug-2025
Accepted:	28-Aug-2025

Senior Editor: Kim Barrett

Reviewing Editor: Kim Barrett

Transaction Report:

(Note: With the exception of the correction of typographical or spelling errors that could be a source of ambiguity, letters and reports are not edited. Depending on transfer agreements, referee reports obtained elsewhere may or may not be included in this compilation. Referee reports are anonymous unless the Referee chooses to sign their reports.)

Dear Dr Keely,

Re: JP-TP-2025-289733 "**Digesting diet and exercise's influence on the microbiome.**" by Simon Keely and Kerith Duncanson

Thank you for submitting your manuscript to The Journal of Physiology. It has been assessed by a Reviewing Editor and by 1 expert referee and we are pleased to tell you that it is acceptable for publication following minor revision.

Please advise your co-author of this decision as soon as possible.

The review comments are copied at the end of this email.

Please address all the points raised and incorporate all requested revisions or explain in your Response to Referees why a change has not been made. We hope you will find the comments helpful and that you will be able to return your revised manuscript within 2 weeks. If you require longer than this, please contact journal staff: jp@physoc.org.

Your revised manuscript should be submitted online using the link in your Author Tasks: Link Not Available. This link is accessible via your account as Corresponding Author; it is not available to your co-authors. If this presents a problem, please contact journal staff (jp@physoc.org). Image files from the previous version are retained on the system. Please ensure you replace or remove any files that are being revised.

If you do not wish to submit a revised version of your manuscript, you must inform our journal staff (jp@physoc.org) or reply to this email to request withdrawal. Please note that a manuscript must be formally withdrawn from the peer review process at one journal before it may be submitted to another journal.

TRANSPARENT PEER REVIEW POLICY: To improve the transparency of its peer review process, The Journal of Physiology publishes online as supporting information the peer review history of all articles accepted for publication. Readers will have access to decision letters, including Editors' comments and referee reports, for each version of the manuscript, as well as any author responses to peer review comments. Referees can decide whether or not they wish to be named on the peer review history document.

LANGUAGE EDITING AND SUPPORT FOR PUBLICATION: If you would like help with English language editing, or other article preparation support, Wiley Editing Services offers expert help, including English Language Editing, as well as translation, manuscript formatting, and figure formatting at www.wileyauthors.com/eeo/preparation. You can also find resources for Preparing Your Article for general guidance about writing and preparing your manuscript at www.wileyauthors.com/eeo/prepresources.

REVISION CHECKLIST: Upload a full Response to Referees file. To create your 'Response to Referees' copy all the reports, including any comments from the Senior and Reviewing Editors, into a Microsoft Word, or similar, file and respond to each point, using font or background colour to distinguish comments and responses and upload as the required file type.

Please upload two versions of your manuscript text: one with all relevant changes highlighted and one clean version with no changes tracked. The manuscript file should include all tables and figure legends, but each figure/graph should be uploaded as separate, high-resolution files.

You may also upload:

- 'Potential Cover Art' for consideration as the issue's cover image
- Appropriate Supporting Information (video, audio or data set: see https://jp.msubmit.net/cgi-bin/main.plex?form_type=display_requirements#supp).

We look forward to receiving your revised submission.

If you have any queries, please reply to this email and we will be pleased to advise.

Best wishes,

Kim Barrett
Senior Editor
The Journal of Physiology

EDITOR COMMENTS

Reviewing Editor:

Dear Simon,

Many thanks for writing this thoughtful Translational Perspectives article. I agree with the authors that your commentary, including the caveats discussed in relation to the findings of the original manuscript, adds important additional context that will be of interest to the readers.

My only very minor comment would be that the title is a little confusing as written and perhaps consider changing it to "Digesting the influence of diet and exercise on the microbiome"

Senior Editor:

Thanks for this excellent piece, Simon. Just let me know if you want to change the title, as suggested by Stephen.

REFEREE COMMENTS

Referee #1:

This is an interesting perspective article that makes useful and valuable comments regarding our recent paper. I imagine that readers will benefit from reading this work as part of the wider context for these new findings.

END OF COMMENTS

From: Simon Keely <simon.keely@newcastle.edu.au>
Sent: 25 August 2025 03:23
To: The Journal of Physiology <jp@physoc.org>
Subject: RE: JP-TP-2025-289733 - The Journal of Physiology - Decision Letter

Hi Kim,

Thank you! Very happy to change the title as suggested by Stephen.

I appreciate the opportunity for the piece.

Kindest Regards

Simon

Dear Simon,

Re: JP-TP-2025-289733R1 "**Digesting the influence of diet and exercise on the microbiome**" by Simon Keely and Kerith Duncanson

We are pleased to tell you that your paper has been accepted for publication in The Journal of Physiology.

TRANSPARENT PEER REVIEW POLICY: To improve the transparency of its peer review process, The Journal of Physiology publishes online as supporting information the peer review history of all articles accepted for publication. Readers will have access to decision letters, including Editors' comments and referee reports, for each version of the manuscript, as well as any author responses to peer review comments. Referees can decide whether or not they wish to be named on the peer review history document.

The last Word (or similar) version of the manuscript provided will be used by the Production Editor to prepare your proof. When this is ready you will receive an email containing a link to Wiley's Online Proofing System. The proof should be thoroughly checked and corrected as promptly as possible.

Authors should note that it is too late at this point to offer corrections prior to proofing. Major corrections at proof stage, such as changes to figures, will be referred to the Editors for approval before they can be incorporated. Only minor changes, such as to style and consistency, should be made at proof stage. Changes that need to be made after proof stage will usually require a formal correction notice.

All queries at proof stage should be sent to: TJP@wiley.com.

Are you on Twitter? Once your paper is online, why not share your achievement with your followers? Please tag The Journal (@jphysiol) in any tweets and we will share your accepted paper with our 30,000 followers!

If you would like to receive our 'Research Roundup', a monthly newsletter highlighting the cutting-edge research published in The Physiological Society's family of journals (The Journal of Physiology, Experimental Physiology and Physiological Reports), please click this link, fill in your name and email address and select 'Research Roundup': https://www.physoc.org/journals-and-media/membernews/

Best wishes,

Kim Barrett
Senior Editor
The Journal of Physiology

EDITOR COMMENTS

Thanks so much for your thoughtful contribution, Simon!

P.S. - You can help your research get the attention it deserves! Check out Wiley's free Promotion Guide for best-practice recommendations for promoting your work at www.wileyauthors.com/eeo/guide. You can learn more about Wiley Editing Services which offers professional video, design, and writing services to create shareable video abstracts, infographics, conference posters, lay summaries, and research news stories for your research at www.wileyauthors.com/eeo/promotion.

IMPORTANT NOTICE ABOUT OPEN ACCESS: To assist authors whose funding agencies mandate public access to published research findings sooner than 12 months after publication, The Journal of Physiology allows authors to pay an Open Access (OA) fee to have their papers made freely available immediately on publication.

The Corresponding Author will receive an email from Wiley with details on how to register or log-in to Wiley Authors Services where you will be able to place an order.

You can check if your funder or institution has a Wiley Open Access Account here: https://authorservices.wiley.com/author-resources/Journal-Authors/licensing-and-open-access/open-access/author-compliance-tool.html.
